# Factors Influencing Quality of Care Service of Caregivers for Preschoolers

**DOI:** 10.3390/ijerph18084291

**Published:** 2021-04-18

**Authors:** Soyeon Jung, Younhee Hong, Sohyune Sok

**Affiliations:** 1 Department of Nursing, Kyung Hee University, Seoul 02447, Korea; semorf@hanmail.net (S.J.); yuni3000@naver.com (Y.H.); 2 College of Nursing Science, Kyung Hee University, Seoul 02447, Korea

**Keywords:** quality of care service, caregiver, preschooler, interaction

## Abstract

The number of households that have benefited from childcare support services has steadily increased, while the number of caregivers has also climbed. Studies on the care service of childcare providers are needed. This study was to examine and identify the factors influencing the degree of quality of care service for preschoolers among caregivers. A cross-sectional descriptive design was employed. Samples included 138 caregivers for preschoolers in health family support centers, Gyeonggi-do, South Korea. Data included the general characteristics of study participants, quality of care service for preschoolers, childcare efficacy, job satisfaction, and caregiver–child interaction. Data were collected from June to August 2019. The factors influencing the quality of care service for preschoolers among caregivers were caregiver–child interaction. The explanatory power of the final regression model was 37%. This study suggests that the caregiver–child interaction should be improved to improve the quality of care service for preschoolers among caregivers, and health professionals need to pay attention to this issue in community fields. The findings from this study can be implied on health policy for childcare support service.

## 1. Introduction

With the growing presence of professional women in society coupled with the continuously shrinking size of the average family, childminding support provided by family members has steadily declined [1]. Working moms and fathers are having difficulties raising their children due to various issues, such as early and late work hours, holiday work, emergency situations, and parental participation in childcare and educational institutions [2]. The childcare support project, where caregivers visit families with children under the age of 12 to take care of them, was initiated as a response to the temporary care demands arising from overtime work and children becoming ill [3]. The project was also aimed at meeting the demand for individual home care. It was designed to promote the welfare of the children, improve the quality of life of family members by ensuring work–home balance for the parents, as well as to foster a child-friendly social environment. Since its implementation in 2007, the number of households that have benefited from the service has steadily increased to 39,138 in 2011 and 64,591 in 2018, while the number of caregivers has also climbed [3].

Public interest in the quality of childcare support programs and childcare providers has increased as well with the expansion in the quantity of childcare support. In particular, the quality of care services provided by caregivers not only ensures user satisfaction but also helps achieve family well-being and family development by allowing the parents to entrust their children to caregivers confidently. In addition, 71.4% of 87,131 children entrusted to part-time care services in 2017 were under 6 years of age, while 100% of children entrusted to all-day care programs were under 36 months of age, suggesting that the majority of childcare service recipients were those less than 6 years of age and preschool-age children [4]. The preschool stage, including infancy, is the foundation of human development, highlighting the growing importance of ensuring the quality of care provided to preschool children by caregivers who take care of children at home on behalf of the parents [5].

Many variables that affect the quality of care service have been reported in previous studies. Among them, the perceived efficacy of childcare is based on how it can induce the development of children towards the direction intended by the teacher in the field of childcare [6]. It was reported that the perceived efficacy influences the interaction with infants [5] and that higher perceived efficacy results in higher overall quality of teacher behavior [7]. This suggests that caregivers provide childcare services, including in-home care, while the perceived efficacy of caregivers, i.e., the belief that caregivers can steer children into intended directions, may affect the quality of care services. Job satisfaction refers to a positive emotional state in which an individual is satisfied with their job. It has been singled out as a major factor affecting the quality of services in a number of studies of nursing caregivers and social workers who provide welfare services [8,9,10,11,12]. Since this can be seen as a key indicator of job-related qualitative ability improvement, the job satisfaction of caregivers is also expected to affect the quality of care services.

On the other hand, the preschool children in this study refer to those less than 6 years of age. Since children at this age learn and develop through continuous interaction with the surrounding environment and with their parents, childcare providers, caregivers are key to the early development of children [13]. Caregiver–child interaction is a concept based on teacher–infant interaction [5]. It can be understood as a form of communication between caregivers and preschool children, which also encompasses the overall attitudes and behaviors of caregivers toward children in the care process [4]. Caregivers continuously interact with children throughout all care processes, including childcare and play. Advance studies have also emphasized the importance of focusing on the interaction involved in providing care in order to improve the quality of care by caregivers [4,5,13]. Therefore, it is necessary to carefully examine the interaction between caregivers and preschool children in order to improve the quality of childcare services.

Previous studies on the quality of childcare services have focused on what individual caregivers feel in relation to their jobs, such as role stress, emotional labor, job satisfaction, and resilience [1,9,14], thereby failing to consider the interactions with children who are eligible for care services. It was also difficult to find particular studies that included childcare efficacy, job satisfaction, and caregiver–child interactions to confirm the impact on the quality of care services.

In this study, key variables that explain the quality of care services have been narrowed down to the efficacy of childcare, job satisfaction, and caregiver–child interaction, which are the preceding variables that are likely to affect the quality of care services for preschool children. This is in line with the attempt to provide basic data for preparing practical and policy measures that are required to identify and improve the quality of care services. The purpose of this study was to examine and identify the factors influencing the degree of quality of care service of caregivers for preschoolers. The aims of the study were: (1) to identify the general characteristics of caregivers for preschoolers; (2) to examine the degree of quality of care service for preschoolers and factors related to it; (3) to examine the differences of the degrees of quality of care service for preschoolers according to the general characteristics of caregivers; (4) to examine the correlations between quality of care service for preschoolers and factors related to it; (5) to determine the factors that influence the degree of quality of care service for preschoolers among caregivers.

## 2. Material and Methods

### 2.1. Study Design and Participants

A cross-sectional descriptive design was employed. Participants included 138 female caregivers for preschoolers in health family support centers, Gyeonggi-do, South Korea. In Korean culture, most of the people caring for preschoolers are women. Study participants participated through convenience sampling in this study. The eligibility criteria were: consented to participate in this study, understood the purpose of this study, had the capability to understand Korean verbally. Of the 150 questionnaires, 145 (96.7%) were answered. Due to incomplete data, only a total of 138 questionnaire responses were included in the final dataset. Sample size adequacy (*n* = 119) using G power 3 analysis software was estimated based on an alpha level = 0.05, medium effect size = 0.15, and power = 0.95 [15]. The sample size of the study was adequate.

### 2.2. Measures

The study participant’s general characteristics survey was developed by researchers based on a literature review and previous research [1,9,13,14,16,17,18], a set of general characteristic variables, including age, educational background, child-rearing experience, duration of work with caregivers, type of caregiver activity, work hours per week, satisfaction with salary levels, relationship with users (guardians), relationship with beneficiaries (children), and appropriateness of care environment were obtained via self-reporting. This consisted of a total of 10 items. 

The childcare efficacy scale developed by Hyeon [6] and then modified by Kang and Kwon [16] for caregivers was used in this study to measure the perceived childcare efficacy of preschool children’s caregivers. A total of 14 questions in the 5-point Likert scale were devised: ‘Strongly disagree (1 point)’, ‘Disagree (2 points)’, ‘Neither agree nor disagree (3 points)’, ‘Agree (4 points)’, ‘Strongly agree (5 points)’. The score ranged from 14 to 70 points, and the higher the score, the higher the caregivers’ perceived efficacy. The childcare efficacy scale showed an acceptable to good correlation and content validity in a previous study [16]. At the time of the modification [16], Cronbach’s α = 0.87, and reliabilities in this study were Cronbach’s α = 0.83.

The job satisfaction scale developed by Bae [19] and then modified by Yoo [18] for caregivers was used in this study to measure the job satisfaction of preschool children’s caregivers. A total of 9 questions in the 5-point Likert scale were devised: ‘Strongly disagree (1 point)’, ‘Disagree (2 points)’, ‘Neither agree nor disagree (3 points)’, ‘Agree (4 points)’, ‘Strongly agree (5 points)’. The score ranged from 9 to 45 points, and the higher the score, the higher the caregivers’ job satisfaction. As regarding the *job satisfaction* scale, at the time of the revision [18], Cronbach’s α = 0.90. In this study, Cronbach’s α = 0.85.

As a tool to measure the interaction between caregivers for preschool children and children, caregivers–child interaction scale developed by Kang and Paik [20] for caregivers and infants was used in this study. In the study of Kang and Paik [20], infants refer to preschool children under the age of 6 according to Chapter 1, Article 2 of the Child Care Act. The tool consisted of a total of 30 questions that are broken down into three sub-groups, each of 10 questions—emotional interactions, verbal interactions, and behavioral interactions. Emotional interaction means interacting with infants through a compassionate and kind attitude, and verbal interaction means verbally interacting with infants’ thoughts, feelings, requests, and expressions, whereas behavioral interaction refers to behaviorally interacting with infants’ appropriate and inappropriate behaviors. Among the questions on behavioral interaction, the ‘classroom’ in ‘Let the classroom have pleasant conversations and laughter’ was modified as a ‘caring environment’ to suit the purpose of this study better. This survey scale consisted of a total of 30 questions in a 5-point Likert method: ‘Strongly disagree (1 point)’, ‘Disagree (2 points)’, ‘Neither agree nor disagree (3 points)’, ‘Agree (4 points)’, ‘Strongly agree (5 points)’. The score ranged from 30 to 150 points, and the higher the score, the better the caregiver–child interaction is. Concerning the caregiver–child interaction scale, in Kang and Paik’s study [20], Cronbac’s α = 0.91 was reported for emotional interaction, Cronbach’s α = 0.88 for verbal interaction, and Cronbach’s α = 0.86 for behavioral interaction. However, in this study Cronbach’s α = 0.90 was reported for emotional interaction, Cronbach’s α = 0.86 for verbal interaction and Cronbach’s α = 0.88 for behavioral interaction.

The ‘SERVQUAL’ scale, first developed by Parasuraman et al. [21] and then modified by Kim and Choi [14] for caregivers and social service workers, was used to measure the quality of care services of preschool children’s caregivers. It consists of a total of 20 questions, 4 questions for each of the 5 sub-areas: reliability, responsiveness, confidence, empathy, and tangibility. Specifically, 4 questions fall in the reliability area, such as, ‘I must provide the originally planned service’, 4 questions in the responsiveness area, such as, ‘I respond according to the user’s needs’, and another 4 questions in the confidence area, such as, ‘I make the user feel secure in the service’, 4 questions in the empathy area, such as, ‘I try to understand the emotions of the users’, and finally 4 questions in the tangible area, such as, ‘I try to give the user a neat and clean impression.’ This scale is a 5-point Likert scale: ‘Strongly disagree (1 point)’, ‘Disagree (2 points)’, ‘Neither agree nor disagree (3 points)’, ‘Agree (4 points)’, ‘Strongly agree (5 points)’. The higher the score, the higher the quality of care service provided by the caregiver is evaluated. In the Korean version of the SERVQUAL scale, the scale presented an acceptable content validity in a previous study [14]. At the time of the development to the Korean version [14], Cronbach’s α = 0.92, and reliabilities of the scale in this study, Cronbach’s α = 0.91.

### 2.3. Procedures

Data were collected from June to August 2019. Researchers contacted the prospective caregivers for preschoolers to explain the purpose of this study, as well as participation details and questionnaire used. Researchers received written informed consent forms from the caregivers who agreed to participate in this study. The questionnaires were given only to caregivers who agreed to participate in the study. After that, the completed questionnaires were collected. The survey consisted of a self-reporting questionnaire managed by researchers. Each questionnaire took about 20–25 min to complete. 

### 2.4. Statistical Analysis

Data were analyzed by SPSS PC+ version 24.0 statistical software program. The general characteristics of the study participants and levels of study variables were analyzed using descriptive statistics. Differences in the degrees of quality of care service for preschoolers according to the general characteristics of the study participants were analyzed using t-test, ANOVA with Scheffe post hoc test. Correlations between the quality of care service for preschoolers and related factors were analyzed using Pearson’s correlation coefficient. Multiple regression analysis was used to examine the factors affecting the quality of care service for preschoolers. A *p*-value of less than 0.05 was considered statistically significant.

### 2.5. Ethical Considerations 

The Institutional Review Board of K University, Seoul, Korea approved this study (IRB No. KHSIRB-19-101(RA)). After the IRB approval, the researchers visited the health family support centers, explained the purpose and procedure of the study, and obtained the approval for data collection. We revealed that the data would not be used for purposes other than research by obtaining voluntary cooperation on data collection from the subjects. In addition, the subjects were informed that they could withdraw from the study at any time. They were also informed of the anonymity and confidentiality of the data obtained from them. The researchers obtained completed written consent forms from eligible subjects prior to their participation.

## 3. Results

### 3.1. General Characteristics of the Study Participants

All participants were female. The average age was 56.88 years old, and 50 to 60 years old or younger was the biggest age group at 53.6%. As for the educational background, most of them were high school graduates (59.4%). For child-rearing experience, 94.9% were experienced. As for the duration of work with caregivers, the average working period was 3.93 years, and more than 3 years and less than 5 years was the biggest group at 24.6%, followed by the group with more than 7 years at 23.2%. Most of the participants were working part-time (95.7%). In regard to work hours per week, the average was 20.97 hours per week, and the group with less than 20 hours was the biggest at 42.0%. As for salary satisfaction, most of the participants were moderate (55.1%). In regard to the relationship with children, most of the participants responded “Good” or “Very good” (87.7%). As for the appropriateness of the care environment, 78.3% of them answered “Moderate” or “good” (Table 1).

### 3.2. Levels of Quality of Care Service for Preschoolers and Related Factors in Caregivers

The mean score for quality of care service was 84.20, which indicates a high quality of care service when compared to the median value (60 points). The mean score of childcare efficacy was 53.90, which indicates a high childcare efficacy when compared to the median value (42 points). The mean score for job satisfaction was 34.56, which indicates a high job satisfaction when compared to the median value (27 points). The mean score for caregiver–child interaction was 127.80, which indicates a high caregiver–child interaction when compared to the median value (90 points) (Table 2).

### 3.3. Differences of Quality of Care Service for Preschoolers according to General Characteristics of Caregivers

There were differences in the means score of quality of care service in some of the participant characteristics related to: the relationship with users (guardians) (F = 15.97, *p* < 0.001), relationship with beneficiaries (children) (F = 5.07, *p* = 0.002), and appropriateness of care environment (F = 3.94, *p* = 0.010). As a result, it was found that the caregiver’s relationship with the user (guardians) was very good, the relationship with the beneficiary (children) was very good, and the caregivers who had very good suitability in the care environment had a higher quality of care service (Table 1).

### 3.4. Correlations between Quality of Care Service for Preschoolers and Related Factors 

Quality of care service had significant, positive relations with relationship with users (guardians) (γ = 0.289, *p* < 0.01), relationship with beneficiaries (children) (γ = 0.262, *p* < 0.01), appropriateness of care environment (γ = 0.252, *p* < 0.01), childcare efficacy (γ = 0.447, *p* < 0.001), job satisfaction (γ = 0.246, *p* < 0.01), and caregiver–child interaction (γ = 0.616, *p* < 0.001) at a statistical significance level of *p* < 0.05 (Table 3).

### 3.5. Factors Influencing Quality of Care Service for Preschoolers

The test that was conducted on the assumptions of the regression analysis showed that all the assumptions coincided with the required assumptions of the regression equations. First, there was no multicollinearity problems (Durbin–Watson value = 1.815; tolerance limit = 0.49–0.72; Variance Inflation Factor (VIF) = 1.38–2.02). All the independent variables were established to be independent of one another (correlations among study variables: from 0.246 to 0.616). For the assumption of the linearity model, the normality of the error term and homoscedasticity were satisfactory.

Based on the results, multiple-regression analyses of relationship with users (guardians), relationship with beneficiaries (children), appropriateness of care environment, childcare efficacy, job satisfaction, and caregiver–child interaction of the study participants were performed to identify the major factors influencing the degree of quality of care service in caregivers for preschoolers. The analyses showed that the prediction model of quality of care service in caregivers for preschoolers was significant (F = 14.11, *p* < 0.001). The value of the adjusted R^2^ was 0.37, which corresponds to the explanatory power of 37%. The factor that was found to have the most influence on the degree of quality of care service in caregivers for preschoolers was the caregiver–child interaction (β = 0.56, *p* < 0.001) (Table 4). 

## 4. Discussion

With regard to the general characteristics of the preschool children’s caregivers in this study, the average age and the educational level were also similar to the previous studies [4,21]. As for the employment type, ‘part-time’ accounted for the majority with 95.7%, which was somewhat higher than the part-time service of 84.0% reported in the 2018 childcare service survey [4]. This is attributed to the fact that in one area where the survey subjects provide services, beneficiaries with children under 36 months of age prefer part-time services along with institutional care, such as daycare, rather than family-based care through full-day services.

In this study, the quality level of care services of preschool children was somewhat higher than that of previous studies [1,9,14]. This is attributed to the fact that the study subjects perceived the quality of care services higher than those of other studies. The perceived childcare efficacy level of preschool children’s caregivers was similar to that of previous studies targeting caregivers [13,18]. Job satisfaction level was also similar to that of a previous study targeting caregivers [18]. The interaction degree between caregivers and children was similar to the teacher–infant interaction degree in the previous study [20].

The relationships with the caring child and guardians and the appropriateness of the caring environment showed significant statistical differences in the quality of care services among the groups in this study and a significant positive correlation with the quality of care service. However, there was no significant influence on the quality of care services. In previous studies [8,22] on work environment and service quality, most of the work environments, including institutional stability, work environment, financial compensation, workload, and use vacation time, differed from the work environment factors considered in this study. This suggests the need for repeat studies on the relationship among the children, the care environment, and the quality of care services.

The perceived childcare efficacy of preschool children’s caregivers showed a positive correlation with the quality of care services, and this was similar to the findings of previous studies [5,23]. However, no direct influence from the perceived childcare efficacy on the quality of care services was reported in this study. This difference seems to be stemming from the work characteristics unique to the study subjects. First of all, 95.7% of the subjects of this study provided part-time care services, with their average weekly work hours logged at 20.97 hours. Part-time care services include schools, childcare facilities, subsidiary of the house and supplies, temporary childcare before the parents arrive home, play activities, and provision of prepared meals and snacks, prompting us to conclude that childcare does not account for a large share of care services within a limited time.

The job satisfaction of preschool children’s caregivers shows a significant positive correlation with the quality of care services, and this is consistent with previous studies [9,10,11] that demonstrated job satisfaction has a positive effect on service quality. However, it was reported in this study that there was no direct influence on the quality of care services, and this result somewhat differs from previous studies. In previous studies, the job satisfaction of caregivers had a positive effect on the quality of childcare services [9,10], and the job satisfaction in nursing home care centers that provide services through home visits significantly affected the quality of nursing services [12]. However, in the case of an activity assistant for the disabled who visits homes after completing a certain training course and cares for the disabled, the job satisfaction and compensation had a significant effect on the quality of service among the sub-factors of job satisfaction. The workload, working environment, and social perception on job satisfaction seem to have no influence on the quality of service [24], providing partial support to the findings of this study. In Kim and Lee’s (2015) study of caregivers, however, it was reported that job satisfaction of caregivers affects service quality and has a mediating effect on the relationship between role stress and service quality of caregivers. Kim and Lee’s study [9] showed some differences from this study in terms of age distribution, caregiver careers, and quality of care services, but such a simple comparison is not likely to be statistically significant. All these highlight the need to further examination of caregivers’ job satisfaction and quality of care services more than once.

Lastly, it was found that the interaction between preschool children and caregivers showed a positive impact on the quality of care services. In other words, better interaction between caregivers and preschool children is likely to result in a higher quality of care services. Compared with previous studies, Um [25], on the other hand, emphasized the quality of the process in which the service is provided since interpersonal care services are provided through interaction between the beneficiaries and the service providers. In addition, Bromer and Korfmacher [26] reported that high-quality support service in home-based childcare focus on the interactions between providers and children. A meta-analysis study by Werner et al. [27] explained that interventions focused on the caregiver–child interaction are effective in improving the quality of childcare. Parasuraman et al. [21] suggested that responsiveness, confidence, and empathy among the components of service quality incorporate the interest and consideration that caregivers give, sincere communication, and rapid response to user needs significantly affect quality of care services, similar to what is necessary for caregivers’ interactions with children. These previous studies are consistent with findings that the interaction between caregivers, the service provider, and preschool children is a major factor in the quality of care services. Folbre [28] suggested that caregiving is performed in a one-to-one relationship, and the quality of interaction, including emotional factors, varies considerably. Unlike other caregiving services, the elements of childcare service are a caregiver and a child, prompting us to conclude that each interaction with users is expected to materialize in various ways. This study focused on the interaction between caregivers and preschool children, given that the beneficiaries are in constant contact with caregivers. The findings confirmed that these interactions between caregivers and children serve as the main factor related to the perceived quality of care services by caregivers. In addition, given the findings of the previous studies [29,30], it is believed that the time to care for children and the interaction between caregivers and children is not absolutely proportionally related. Even if the time of care is relatively short, high-quality interactions are possible and are revealed to be a major factor in determining the quality of care services. Therefore, it seems necessary to explore variables related to this so that high-quality interactions between caregivers and children are being achieved.

The findings of this study indicate that better interaction between caregivers and preschool children results in better care services quality. Since caregivers represent a major driving force that helps children in the home, such as health, hygiene, education, and play, the quality of caregivers’ care services not only affect users’ service satisfaction but also affects the healthy growth and development of the children cared for [1,9]. Kim [30] emphasized that in order to improve the quality of care, it is necessary to focus on the qualitative enhancement of childcare services rather than the quantitative expansion through intensive discussions on the quality of care. Based on the results of this study, it is expected that it will be possible to contribute to the quality improvement in care services through high-quality interactions between child caregivers and children, and through this, it is expected to cause a positive effect on childcare and family welfare.

This study can serve as the basis for efforts to improve the quality of care services rendered by caregivers and also offers base data for follow-up research and health policy. In addition, the findings from this study can be applied to health policy for childcare support services. It will also serve as the basis for health policy on children’s health and contribute to community health through healthy family development wherein childcare services are indispensable. Further studies exploring the intervening related factors that affect the quality of care services of caregivers should be continued in order to maintain personal care. The text refers to the care of healthy children without any serious illness. However, it would be necessary to have further studies for children with some degree of disability or special needs. In particular, there development and application of education and intervention programs that can enhance interaction between caregivers and children should be pursued. In addition, more qualitative research is needed on parents’ thoughts and perceptions of the services provided to care for their children to explore the caregiver–child–parent interactions needed to achieve maximum satisfaction.

This study has some limitations. This study targets caregivers who provide care services to preschool children under the age of 6 and who are registered in one institution located in the province of Gyeonggi-do, South Korea. It is difficult to generalize this study’s findings since it targeted caregivers who have agreed to the study. In addition, the sampling was limited to one regional center, and surveys were also within the range of the sampling. There is one additional limitation to the study: the results are applicable to female caregivers only because there were no male caregivers in the sample.

## 5. Conclusions

In conclusion, the interaction between caregivers and children is the most important variable in explaining the quality of care services. Therefore, it is important to increase the interaction between caregivers and children in order to improve the quality of care services provided by caregivers to preschool children.

This study is significant because it deals with the interaction between caregivers and preschool children, which was difficult to find in previous studies. Job satisfaction has been treated as a major variable to explain the quality of service in many studies. In this study, the interaction between caregivers and children rather than job satisfaction was shown to be the most important variable in explaining the quality of care services.

## Figures and Tables

**Table 1:** Quality of care service for preschoolers according to general characteristics of caregivers.

Characteristics	n	%	Quality of Care Service	
			Mean ± SD	t/F (*p*) Scheffe
Age (year)				0.45 (0.638)
<50	13	9.4	4.20 ± 0.32	
50≤ ≤ 60	74	53.6	4.19 ± 0.39	
60<	51	37.0	4.25 ± 0.39	
Mean ± SD	56.88 (5.76)			
Education background				1.18 (0.321)
Elementary school	2	1.4	4.33 ± 0.67	
Middle school	3	2.2	4.42 ± 0.38	
High school	82	59.4	4.19 ± 0.39	
College	21	15.2	4.12 ± 0.28	
University or higher	30	21.7	4.31 ± 0.39	
Child-rearing experience				0.23 (0.819)
Yes	131	94.9	4.21 ± 0.39	
No	7	5.1	4.18 ± 0.35	
Duration of work with caregivers (year)				1.44 (0.226)
<1	24	17.4	4.34 ± 0.41	
1≤ ≤ 3	25	18.1	4.11 ± 0.32	
3< < 5	34	24.6	4.16 ± 0.41	
5≤ ≤ 7	23	16.7	4.18 ± 0.37	
7<	32	23.2	4.26 ± 0.37	
Mean ± SD	3.93 (3.02)			
Type of caregiver activity				0.64 (0.526)
Part-time	132	95.7	4.21 ± 0.38	
Infant full-time	2	1.4	4.50 ± 0.28	
Combined	4	2.9	4.14 ± 0.38	
Work hours per week (hours)				0.38 (0.685)
<20	58	42.0	4.20 ± 0.39	
20≤ ≤ 30	48	34.8	4.25 ± 0.39	
30<	32	23.2	4.17 ± 0.37	
Mean ± SD	20.97 (11.49)			
Satisfaction with salary levels				0.80 (0.529)
Very satisfied	2	1.4	4.08 ± 0.25	
Satisfied	25	18.1	4.29 ± 0.36	
Moderate	76	55.1	4.19 ± 0.37	
Dissatisfied	28	20.3	4.16 ± 0.45	
Very dissatisfied	7	5.1	4.37 ± 0.37	
Relationship with user (guardians)				15.97 (< 0.001) * a < b
Moderate	19	13.8	4.23 ± 0.44 ^a^	
Good	83	60.1	4.09 ± 0.30 ^a^	
Very good	36	26.1	4.48 ± 0.39 ^b^	
Relationship with beneficiaries (children)				5.07 (0.002) * a < b
Bad	3	2.2	3.90 ± 0.13 ^a^	
Moderate	14	10.1	4.19 ± 0.44 ^ab^	
Good	80	58.0	4.14 ± 0.31 ^ab^	
Very good	41	29.7	4.39 ± 0.44 ^b^	
Appropriateness of care environment				3.94 (0.010) * a < b
Bad	7	5.1	3.94 ± 0.41 ^a^	
Moderate	35	25.4	4.17 ± 0.31 ^ab^	
Good	73	52.9	4.19 ± 0.35 ^ab^	
Very good	23	16.7	4.42 ± 0.49 ^b^	

* *p* < 0.05.

**Table 2:** Levels of quality of care service for preschoolers and related factors in caregivers.

Variables	Average Rating Mean ± SD	Total Score Average Mean ± SD	Range of Points
Quality of care service	4.21 ± 0.37	84.20 ± 7.41	20–100
Childcare efficacy	3.85 ± 0.37	53.90 ± 5.18	14–70
Job satisfaction	3.84 ± 0.50	34.56 ± 4.51	9–45
Caregiver–child interaction	4.26 ± 0.38	127.80 ± 11.42	30–150

**Table 3:** Correlations between the quality of care service for preschoolers and related factors.

Variables	Quality of Care Service
Age	0.066
Duration of work with caregivers	−0.003
Work hours per week	−0.032
Relationship with user (guardians)	0.289 **
Relationship with beneficiaries (children)	0.262 **
Appropriateness of care environment	0.252 **
Childcare efficacy	0.447 ***
Job satisfaction	0.246 **
Caregiver–child interaction	0.616 ***

** *p* < 0.01; *** *p* < 0.001.

**Table 4:** Factors influencing quality of care service for preschoolers.

Variables	B	S.E.	β	t	*p*	Tolerance	VIF
(Constant)	1.40	0.32		4.32	<0.001		
Relationship with user (guardians)	0.04	0.06	0.07	0.77	0.442	0.54	1.85
Relationship with beneficiaries (children)	−0.04	0.05	−0.07	−0.76	0.446	0.72	1.38
Appropriateness of care environment	0.00	0.04	0.00	0.05	0.964	0.55	1.82
Childcare efficacy	0.13	0.10	0.12	1.33	0.184	0.56	1.80
Job satisfaction	−0.03	0.06	−0.04	−0.55	0.580	0.49	2.02
Caregiver–child interaction	0.57	0.09	0.56	6.13	<0.001 *	0.72	1.40
Durbin–Watson = 1.815 (1.632 ≤ d ≤ 1.813), R^2^ = 0.39, Adj. R^2^ = 0.37, F = 14.11, *p* < 0.001 *							

B = Unstandardized coefficients; S.E = Standard Error; ß = Standardized coefficients; t = *t*-test; *p* = *p* value; * *p* < 0.05.

## Data Availability

No new data were created or analyzed in this study. Data sharing is not applicable to this article.
